# Spontaneous vascular dysfunction in Dahl salt‐sensitive male rats raised without a high‐salt diet

**DOI:** 10.14814/phy2.16165

**Published:** 2024-07-24

**Authors:** Arturo Grano de Oro, Sanjana Kumariya, Blair Mell, Jasenka Zubcevic, Bina Joe, Islam Osman

**Affiliations:** ^1^ Department of Physiology and Pharmacology, Center for Hypertension and Personalized Medicine University of Toledo, College of Medicine and Life Sciences Toledo Ohio USA

**Keywords:** animal models of hypertension, endothelial dysfunction, salt‐sensitive hypertension, vascular dysfunction, vascular smooth muscle

## Abstract

Dahl salt‐sensitive (SS) rats fed a high‐salt diet, but not low‐salt, exhibit vascular dysfunction. Several substrains of SS rats exist that differ in their blood pressure phenotypes and salt sensitivity. The goal of this study was to investigate whether the John‐Rapp‐derived SS rat (SS/Jr), which exhibits spontaneous hypertension on a low‐salt diet, presents with hallmarks of vascular dysfunction observed in another experimental model of hypertension independent of dietary salt, the spontaneously hypertensive rat (SHR). Endothelium‐intact aortic rings and mesenteric resistance arteries were isolated from low‐salt fed adult male SS/Jr rats and SHRs, or their respective controls, for isometric wire myography. Vessels were challenged with cumulative concentrations of various vasoactive substances, in the absence or presence of nitric oxide synthase or cyclooxygenase inhibitors. Despite showing some differences in their responses to various vasoactive substances, both SS/Jr rats and SHRs exhibited key features of vascular dysfunction, including endothelial dysfunction and hyperresponsiveness to vasocontractile agonists. In conclusion, this study provides evidence to support the utility of the SS/Jr rat strain maintained on a low‐salt diet as a valid experimental model for vascular dysfunction, a key feature of human hypertension.

## 1 INTRODUCTION

Hypertension (HTN) is one of the most common diseases worldwide and is responsible for significant morbidity and mortality. Furthermore, HTN constitutes a major risk factor for heart, kidney, and cerebrovascular complications. Experimental animal models have been useful in understanding the underlying mechanisms of HTN. Their utility relies on their validity in recapitulating features of human HTN including key pathophysiological mechanisms, such as vascular dysfunction (Lerman et al., 2019).

The most commonly studied genetic model of HTN is the spontaneously hypertensive rat (SHR), followed by the Dahl salt‐sensitive (SS) rat (Lerman et al., 2019). SS rats were initially developed by Lewis Dahl from an outbred Sprague Dawley breeding stock with selection for HTN upon high‐salt treatment (i.e., salt sensitivity). Selective breeding of rats that were insensitive to salt led to the development of Dahl salt‐resistant (SR) rats (Dahl et al., 1962). To minimize genetic heterogeneity and blood pressure (BP) variability among the individual SS and SR rats, Rapp and Dene later developed fully inbred SS and SR strains, designated as SS/Jr and SR/Jr, respectively (Rapp & Dene, 1985). Several substrains of the SS/Jr rat strains were later developed by commercial suppliers and academic institutions, such as the SS/JrHsd, SS/JrHsdEnv, SS/JrHsdMcwi, and SS/JrHsdMcwiCrl rat strains. Notably, marked differences exist between the original SS/Jr strain and these derivative substrains (Reviewed in (Rapp & Garrett, 2019)). For instance, when fed a high‐salt diet (8% NaCl), the original SS/Jr rats develop a rapid fulminating HTN after 3–4 weeks and all die within a few months (Rapp & Dene, 1985). Importantly, on a low‐salt diet (0.2%–0.4% NaCl), previous studies (Garrett et al., 2003, 2007; Gillis et al., 2015; Nedvídek & Zicha, 1993; Regner et al., 2012; Terstappen et al., 2019), including from our group (Mell et al., 2015), demonstrate that SS/Jr rats still develop spontaneous age‐related HTN, albeit with a slower progression. Conversely, other substrains, such as the SS/JrHsdMcwi rats, remain normotensive on a low‐salt diet and require a high‐salt diet to elicit a hypertensive response (Cowley Jr. et al., 2016; Endres et al., 2014; Mattson et al., 2008). Consistently, female SS/Jr rats (Gillis et al., 2015), but not SS/JrHsdMcwiCrl (West, 2023), develop spontaneous superimposed preeclampsia while maintained on a low‐salt diet. Using whole genome sequencing, our group has previously reported that the SS/Jr and the SS/JrHsdMcwi rat strains are not genetically equivalent, exhibiting >1.3 million different base pairs that are likely responsible for the reported differences in BP phenotypes (Padmanabhan & Joe, 2017). Consistently, Pai et al. reported genetic differences across SS/JrHsdEnv, SS/JrHsdMcwi, and the original SS/Jr rat strains (Pai et al., 2018).

Early studies have reported vascular functional changes in SS rats fed a high‐salt diet (Boegehold, 1992; Chen & Sanders, 1991, 1993; Hayakawa et al., 1997; Kong et al., 1991; Lüscher et al., 1987; Nishida et al., 1998; Raij et al., 1988; Zhou et al., 1999, 2001), but not on a low‐salt diet (Hayakawa et al., 1997; Kong et al., 1991; Lüscher et al., 1987; Raij et al., 1988; Zhou et al., 1999, 2001). Notably, the SS/Jr substrains used in these previous studies typically required high‐salt feeding to elicit HTN. Importantly, high‐salt feeding per se has become well‐established as an important insult that elicits vascular dysfunction independent of BP or genetic background (Boegehold, 1993; Greaney et al., 2012; Lenda et al., 2000; Li et al., 2009; Lombard et al., 2003; Nurkiewicz et al., 2010; Oberleithner et al., 2007; Zhou et al., 1999; Zhu et al., 2007). Accordingly, the relative contribution of the inherent characteristics/genetics of the SS/Jr rat versus the effects of high‐salt feeding on vascular dysfunction remains unexplored. To address this knowledge gap, in this study, we utilized the SS/Jr rats, which exhibit spontaneous HTN on a low‐salt diet (Garrett et al., 2003, 2007; Gillis et al., 2015; Mell et al., 2015; Regner et al., 2012; Terstappen et al., 2019), and tested whether they exhibit hallmarks of vascular dysfunction while maintained on a low‐salt diet and if so, how they compared with another model of spontaneous hypertension, the SHR strain.

## 2 MATERIALS AND METHODS

### 2.1 Materials

Phenylephrine hydrochloride (PE), 5‐hydroxytryptamine hydrochloride (5‐HT), acetylcholine chloride (Ach), Sodium nitroprusside dihydrate (SNP), Nω‐Nitro‐L‐arginine methyl ester hydrochloride (L‐NAME), Indomethacin, and Terutroban were purchased from Sigma‐Aldrich. (Research Resource Identifier (RRID): SCR_008988). All other chemicals were purchased from Thermo Fisher Scientific (RRID: SCR_008452).

### 2.2 Experimental animals

All animal studies were performed per the University of Toledo Health Science Campus Institutional Animal Care and Use guidelines and were approved by the committee. SS/Jr and SR/Jr inbred rat strains were developed at the University of Toledo College of Medicine and Life Sciences (previously, Medical College of Ohio) and have been maintained in‐house since 1985 (Rapp & Dene, 1985). SS/Jr and SR/Jr rat strains were bred and maintained on a low‐salt diet (0.3% NaCl; Teklad diet 7034, Inotiv), weaned between 28 and 30 days of age, and were euthanized for experiments between 16 and 20 weeks of age, an age where SS/Jr male rats on low‐salt diet are reported to exhibit markedly elevated systolic BP (∼160 mmHg) while SR/Jr male rats remain normotensive (Garrett et al., 2003; Rapp & Dene, 1985).

Adult male SHR rats and their corresponding Wistar Kyoto (WKY) controls were purchased from Charles River laboratories (RRID: SCR_003792), maintained on a standard chow diet (Teklad diet 2916, Inotiv), and were euthanized for experiments between 16 and 20 weeks of age. Reported mean arterial BP for male SHR and WKY rat strains of approximate age to those used in this study were obtained from the rat genome database and are as follows: 151.69 ± 3.57 and 115.25 ± 7.93 mmHg, for SHR and WKY groups, respectively (Smith et al., 2020). Since young adult male rats commonly exhibit higher BP than their female littermates (Sandberg & Ji, 2012), to initially test whether SS/Jr rats maintained on a low‐salt diet may be used as a model of vascular dysfunction, we elected to limit this study to male rats only. Accordingly, male SS/Jr, SR/Jr, SHR, and WKY rats were used in the current study. All rodents were maintained on a 12:12‐hour‐light/dark cycle and were allowed access to both chow and water ad libitum. Euthanasia of rodents was performed by carbon dioxide overdose via a compressed gas cylinder followed by thoracotomy. All euthanasia and tissue harvesting were performed in the Department of Laboratory Animal Resources from 09:00 to 11:00 on experimental days.

### 2.3 Isometric wire myography

Isometric wire myography was performed as previously described (Pyla et al., 2014), with minor changes. Briefly, immediately after animal sacrifice, aortic tissue segments and 3rd‐4th order mesenteric resistance arteries (MRA) were excised and cleaned from the surrounding periadventitial fat and connective tissues in ice‐cold oxygenated modified Krebs bicarbonate (Krebs) buffer (130 mM NaCl, 4.7 mM KCl, 1.17 mM MgSO4, 1.18 mM KH_2_PO4, 1.6 mM CaCl_2_, 25 mM NaHCO_3_, 5.5 mM glucose, and 0.03 mM EDTA; pH 7.4). Krebs buffer was continuously bubbled with a gas mixture of 95% O_2_ and 5% CO_2_.

Aortic tissue segments or MRAs (≈2 mm) were mounted on multi‐chamber Danish Myo Technology pin or wire myographs (DMT 620 M), respectively, and bathed in 37°C oxygenated Krebs buffer. After an initial stabilization period, aortic tissue segments were stretched gradually to a prefixed tension of 10 mN. Conversely, MRAs were stretched gradually to a variable optimum pretension determined using a standard DMT normalization protocol using the following settings: Target pressure = 13.3 Kpa and internal circumference (IC)_1_/IC_100_ = 0.9. After reaching a stable pretension, vessels were incubated for 45 min with multiple wash steps with fresh 37°C oxygenated Krebs buffer.

To test vascular reactivity, endothelium‐intact aortic rings or MRAs were challenged with a high‐KCL solution (120 mM), cumulative concentrations of the vasoconstrictors PE or 5‐HT (10^−9^–10^−4^ M), cumulative concentrations of an endothelial‐dependent vasodilator, Ach (10^−9^–10^−4^ M), or an endothelial‐independent vasodilator, SNP (10^−9^–10^−5^ M). Vessels were washed multiple times between different drug treatments using fresh 37°C oxygenated Krebs buffer till a stable passive basal tension was restored. Relaxation experiments were performed after initial preconstriction using a submaximal concentration of PE (10^−7^ M for aortic rings or 10^−6^ M for MRA). In select experiments, the effects of cumulative concentrations of 5‐HT or Ach were repeated in the presence of a nitric oxide synthase inhibitor, L‐NAME (100 μM, 30 min pretreatment), Indomethacin (10 μM, 30 min pretreatment), or terutroban (100 nM, 30 min pretreatment).

### 2.4 Tissue sectioning and hematoxylin/eosin (HE) staining

Isolated 3rd‐4th order MRA were fixed in 4% paraformaldehyde in PBS over‐night at 4°C, washed three times with PBS, then incubated in 30% sucrose in PBS over‐night at 4°C. Fixed tissues were embedded in optimal cutting temperature compound and kept at −80°C until cryo‐sectioning (8 μm thickness). HE staining was performed following a standard protocol, as previously described (Osman et al., 2019; Osman et al., 2021). HE‐stained images were analyzed for medial thickness and medial cross‐sectional area. The medial cross‐sectional area was calculated by subtraction of the area enclosed by the internal elastic lamina from the area enclosed by the external elastic lamina.

### 2.5 Calculations and statistical analysis

Results are expressed as means ± SD values. The “*N*” value represents the number of animals per group. Contraction in response to high‐KCl solution (10‐min incubation) is expressed in mN. PE or 5‐HT‐induced contractions are expressed as a percentage of the contraction induced by high‐KCl solution. Ach‐ or SNP‐induced relaxations are expressed as a percentage of the precontraction induced by PE (Tables 1 and 2). To determine *E*_max_ and EC_50_ values (−log EC_50_), nonlinear regression analysis was performed using GraphPad Prism software (version 10.2). Statistical analyses of the data among groups were performed by unpaired *t*‐test or two‐way repeated measures ANOVA followed by Fisher's LSD test, as appropriate. Values of *p* < 0.05 were considered statistically significant.

**TABLE 1:** Phenylephrine precontraction data used for Ach and SNP relaxation studies in dorsal aorta.

	SR (mN ± SD)	SS (mN ± SD)	WKY (mN ± SD)	SHR (mN ± SD)
Ach	2.2 ± 1.1	2.4 ± 0.8	6.5 ± 4.1	4.7 ± 3.9
Ach (L‐NAME pretreatment)	6.1 ± 4.5	6.1 ± 1.4	12.5 ± 1.1	7.6 ± 2.3
Ach (L‐NAME + Indomethacin pretreatment)	14.9 ± 11.4	10.3 ± 6		
Ach (L‐NAME + Terutroban pretreatment)	10.6 ± 6	7 ± 1.8		
SNP	2.9 ± 2.2	3.1 ± 1.1		
Ach (Indomethacin pretreatment)			3 ± 0.9	3.9 ± 2.4
Ach (Terutroban pretreatment)			3.9 ± 1.3	12 ± 3.7

**TABLE 2:** Phenylephrine preconstriction data used for Ach and SNP relaxation studies in MRA.

	SR (mN ± SD)	SS (mN ± SD)	WKY (mN ± SD)	SHR (mN ± SD)
Ach	3.6 ± 1.5	6.4 ± 3.6	10.7 ± 3.5	7.9 ± 1.6
Ach (L‐NAME pretreatment)	3.2 ± 1.7	7.8 ± 3.7	11.3 ± 3	7.3 ± 1.8
SNP	7.3 ± 4.9	2.9 ± 0.6		

## 3 RESULTS

### 3.1 Vascular reactivity in aortic rings of SS versus SR male rats

**FIGURE 1:**
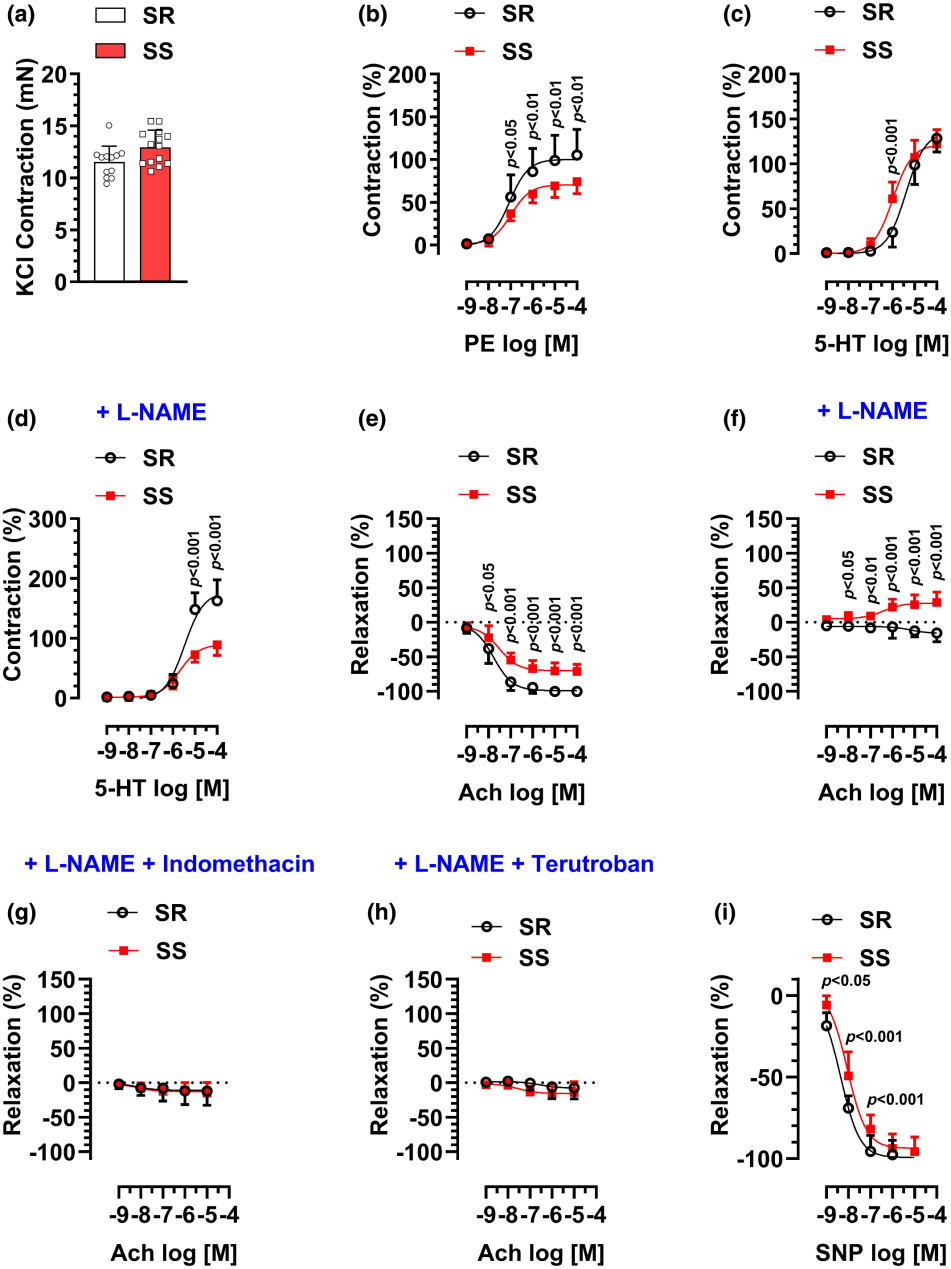
16‐ to 20‐week‐old male Dahl salt‐sensitive (SS) or Dahl salt‐resistant (SR) rats on a low‐salt diet (0.3% NaCl) were sacrificed for vascular reactivity analysis in dorsal aortic rings. (a) Contraction induced by high‐potassium solution (KCl, 120 mM). (b) Contractions induced by phenylephrine (PE, 10^−9^–10^−4^ mol/L [M]). (c) Contractions induced by 5‐hydroxytryptamine (5‐HT, 10^−9^–10^−4^ M). (d) Effects of pretreatment with Nω‐Nitro‐L‐arginine methyl ester (L‐NAME, 100 μM, 30 min) on 5‐HT‐induced contractions. (e) Relaxations induced by acetylcholine (Ach, 10^−9^–10^−4^ M) after precontraction with PE (10^−7^ M). (f) Effects of pretreatment with L‐NAME (100 μM, 30 min) on Ach‐induced relaxation. (g) Effects of pretreatment with L‐NAME (100 μM) plus Indomethacin (10 μM) on Ach‐induced relaxation. (h) Effects of pretreatment with L‐NAME (100 μM) plus Terutroban (100 nM) on Ach‐induced relaxation (i) Relaxations induced by sodium nitroprusside (SNP, 10^−9^–10^−4^ M) after precontraction with PE (10^−7^ M). *N* = 12–14.

High‐KCl solution (120 mM) evoked a strong contraction in aortic rings that was not statistically different between SS/Jr (SS) and SR/Jr (SR) groups (Figure 1a). PE induced a concentration‐dependent contraction in aortic rings in both groups (Figure 1b). However, the effects were significantly lower in the SS group (*E*_max_: 74 ± 14%, 105 ± 30%; EC_50_: −6.98 ± 0.1, −7.07 ± 0.19; for SS and SR groups, respectively).

5‐HT induced a concentration‐dependent contraction in aortic rings in both SS and SR groups (Figure 1c). While there was no difference in the maximum response between both groups, the concentration‐response curve was moderately shifted to the left in the SS group, suggesting enhanced sensitivity (EC_50_: −5.99 ± 0.08, −5.39 ± 0.09, for SS and SR groups, respectively). Notably, L‐NAME pretreatment abolished the observed difference in response to 5‐HT at 10^−6^ M concentration between the SS and SR groups (Figure 1c,d). In addition, L‐NAME pretreatment markedly increased the maximum response to 5‐HT in the SR group but not in the SS group. (*E*_max_: 163 ± 35%, 128 ± 15%, for SR groups, in the presence or absence of L‐NAME, respectively).

In endothelial‐intact aortic rings precontracted with PE, Ach induced a concentration‐dependent relaxation in both SS and SR groups (Figure 1e). However, the relaxation effects were markedly decreased in the SS group (*E*_max_: −71 ± 10%, −100 ± 4%; EC_50_: −7.52 ± 0.15, −7.78 ± 0.12; for SS and SR groups, respectively). As shown in Figure 1f, pretreatment of aortic rings with L‐NAME markedly attenuated Ach‐induced relaxation in the SR group (*E*_max_: −16 ± 13%, −100 ± 4% for the SR group, in the presence or absence of L‐NAME, respectively). However, in the SS group, in the presence of L‐NAME, Ach further enhanced PE‐induced contraction rather than induced relaxation (*E*_max_: 29 ± 15%, −71 ± 10%, for the SS group, in the presence or absence of L‐NAME, respectively). Notably, co‐incubation with L‐NAME plus Indomethacin (a nonselective COX inhibitor) (Figure 1g) or L‐NAME plus Terutroban (a thromboxane/prostaglandin endoperoxide receptor antagonist) (Figure 1h), abolished Ach‐induced contractions in the SS group.

As shown in Figure 1i, in endothelial‐intact aortic rings precontracted with PE, SNP induced a concentration‐dependent relaxation in both SS and SR groups. While there was no difference in the maximum response between both groups, the concentration‐response curve was moderately shifted to the right in the SS group, suggesting decreased sensitivity (EC_50_: −8.05 ± 0.09, −8.36 ± 0.1; for SS and SR groups, respectively).

### 3.2 Vascular reactivity in aortic rings of SHR versus WKY male rats

**FIGURE 2:**
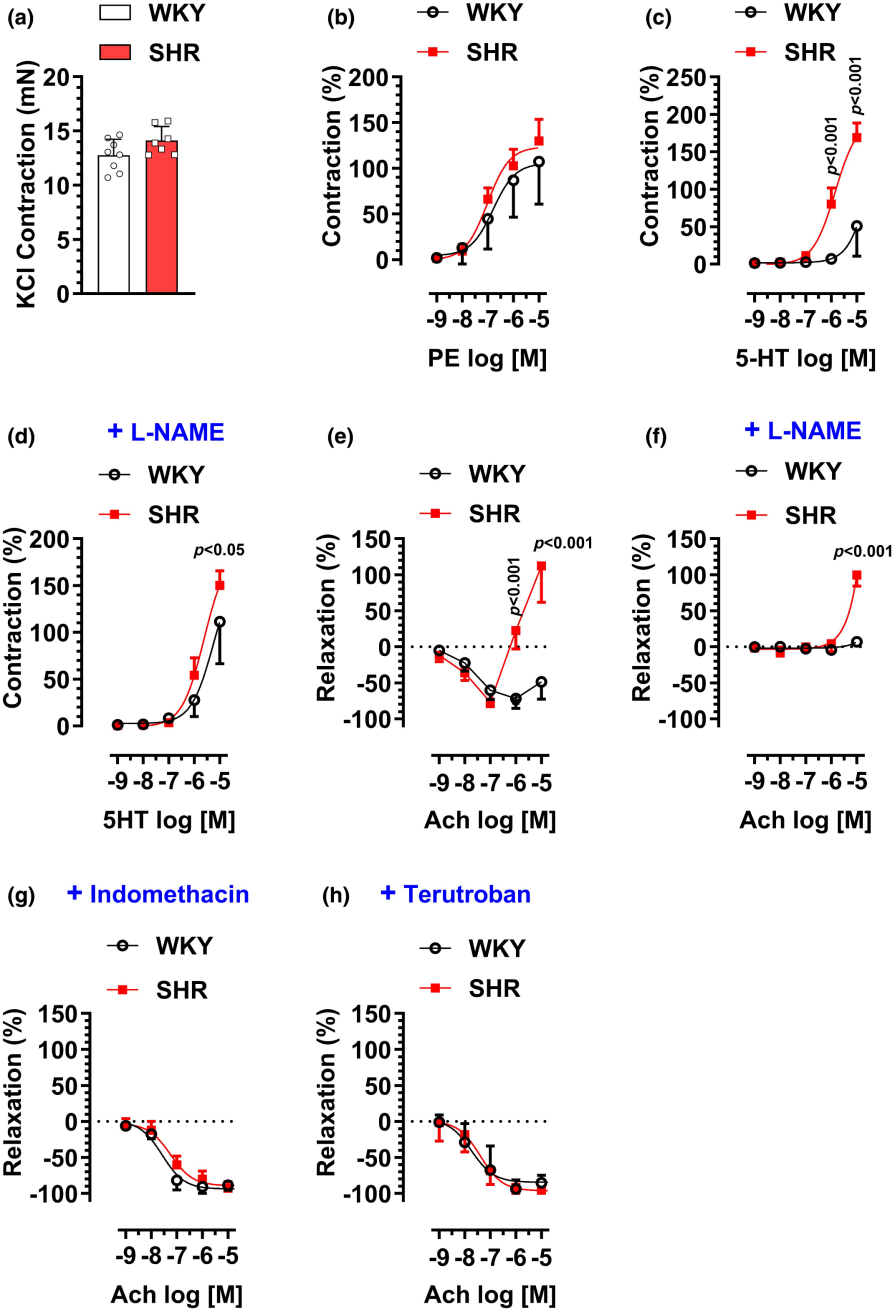
16‐ to 20‐week‐old male spontaneously hypertensive rat (SHR) or Wistar Kyoto (WKY) rats were sacrificed for vascular reactivity analysis in dorsal aortic rings. (a) Contraction induced by KCl (120 mM). (b) Contractions induced by PE (10^−9^–10^−5^ M). (c). Contractions induced by 5‐HT (10^9^–10^−5^ M). (d) Effects of pretreatment with L‐NAME (100 μM, 30 min) on 5‐HT‐induced contractions. (e) Relaxations induced by Ach (10^−9^–10^−5^ M) after precontraction with PE (10^−7^ M). (f). Effects of pretreatment with L‐NAME (100 μM, 30 min) on Ach‐induced relaxation. (g). Effects of pretreatment with Indomethacin (10 μM, 30 min) on Ach‐induced relaxation. (h) Effects of pretreatment with Terutroban (100 nM, 30 min) on Ach‐induced relaxation. *N* = 7–8.

To test whether the SS rats exhibit features of vascular dysfunction similar to those of the SHR strain, we repeated the same vascular reactivity experiments described above using age‐ and sex‐matched SHR and WKY controls. First, we found that high‐KCl solution evoked a strong contraction that was not statistically different between the SHR and WKY groups (Figure 2a). As shown in Figure 2b, PE induced a concentration‐dependent contraction in aortic rings in both groups but exhibited a trend (*p* = 0.07) for an increase in the maximum response in the SHR group (*E*_max_: 130 ± 24%, 107 ± 47%, for SHR and WKY groups, respectively).

As shown in Figure 2c, 5‐HT‐induced contractions in the SHR group were markedly enhanced versus the WKY group (*E*_max_: 169 ± 20%, 51 ± 40%, for SHR and WKY groups, respectively). While L‐NAME pretreatment partly abolished the difference in response to 5‐HT between SHR and WKY groups, 5‐HT still exhibited a higher *E*_max_ in the SHR group in the presence of L‐NAME (*E*_max_: 150 ± 16%, 111 ± 45%, for SHR and WKY groups, in the presence of L‐NAME, respectively) (Figure 2c,d).

As shown in Figure 2e, in endothelial‐intact aortic rings precontracted with PE, increasing concentrations of Ach induced a biphasic response in both SHR and WKY groups. In the SHR group, at lower concentrations, Ach induced relaxation that reached a maximum of −77 ± 17% at 10^−7^ M concentration. However, at higher concentrations, Ach induced marked contraction that even surpassed the precontraction level and reached a maximum of 112 ± 50% at 10^−5^ M concentration. In the WKY group, at concentrations up to 10^−6^ M, Ach induced a concentration‐dependent relaxation that reached a maximum of −72 ± 14%. However, at the maximum concentration used of 10^−5^ M, Ach induced moderate contraction that partly attenuated the initial relaxation response to −49 ± 9%. While pretreatment of aortic rings with L‐NAME almost completely abolished Ach‐induced relaxation in both SHR and WKY groups, it did not alter the marked contraction observed at 10^−5^ M Ach concentration in the SHR group (*E*_max_: 100 ± 15%, 112 ± 50%, for SHR groups, in the presence or absence of L‐NAME, respectively) (Figure 2f). Notably, pretreatment of aortic rings with Indomethacin (Figure 2g) or Terutroban (Figure 2h), abolished Ach‐induced contractions in both SHR and WKY groups. This was accompanied by enhanced Ach‐induced relaxation in both SHR and WKY groups.

### 3.3 Vascular reactivity in mesenteric resistance arteries of SS versus SR male rats

**FIGURE 3:**
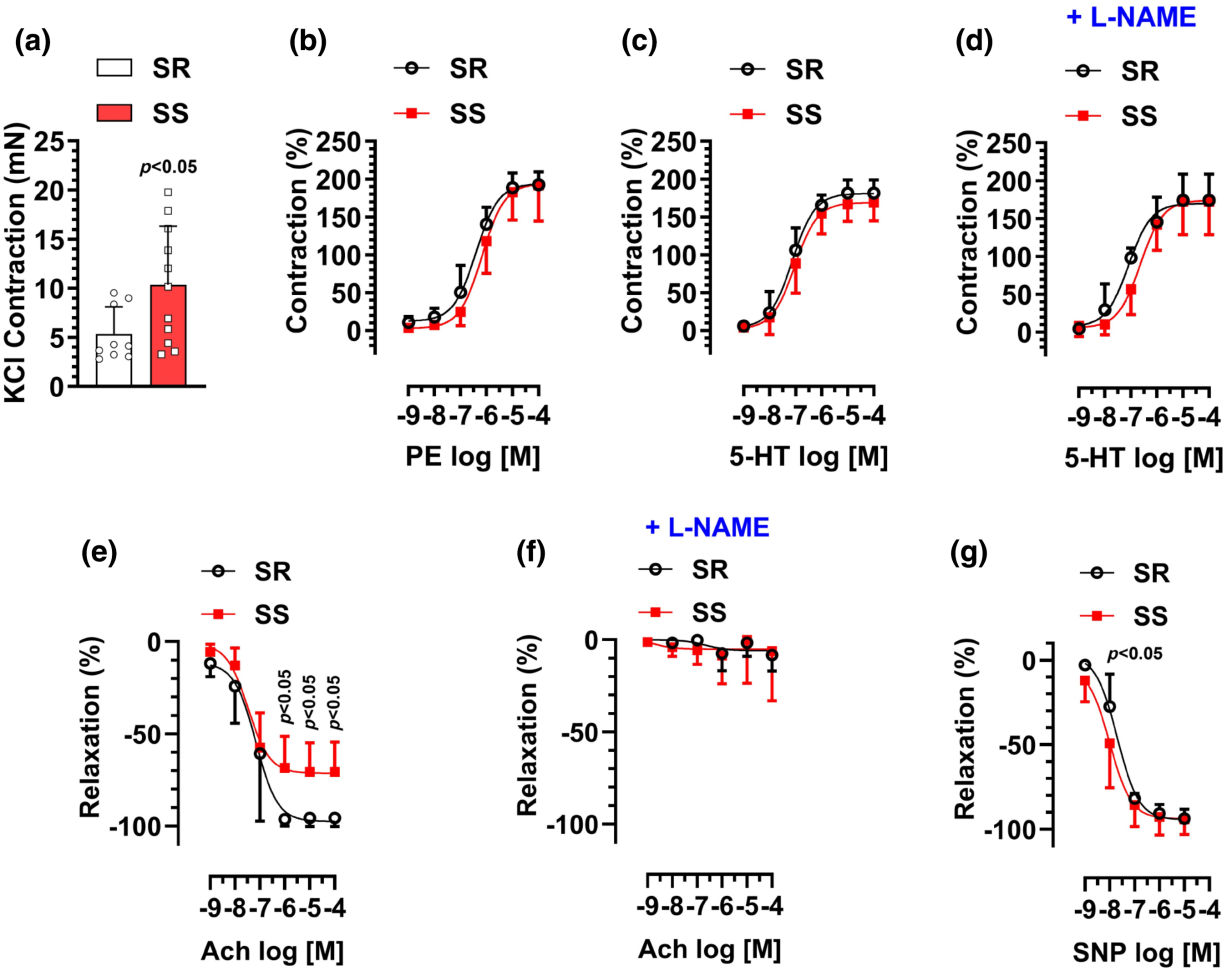
16‐ to 20‐week‐old male SS or SR rats on a low‐salt diet (0.3% NaCl) were sacrificed for vascular reactivity analysis in mesenteric arterial rings. (a) Contraction induced by KCl (120 mM). (b) Contractions induced by PE (10^−9^–10^−4^ M). (c) Contractions induced by 5‐HT (10^9^–10^−4^ M). (d) Effects of pretreatment with L‐NAME (100 μM, 30 min) on 5‐HT‐induced contractions. (e) Relaxations induced by Ach (10^−9^–10^−4^ M) after precontraction with PE (10^−6^ M). (f) Effects of pretreatment with L‐NAME (100 μM, 30 min) on Ach‐induced relaxation. (g) Relaxations induced by SNP (10^−9^–10^−4^ M) after precontraction with PE (10^−6^ M). *N* = 9–11.

Next, we tested the vascular responses in MRAs in SS and SR groups. Despite considerable intragroup variability in response to high‐KCl solution, especially in the SS group, the average response to high‐KCl was almost doubled in the SS group (10.3 ± 5.9 mN, 5.3 ± 7.7 mN, for SS and SR groups, respectively) (Figure 3a). When normalized to high‐KCl responses, both PE‐ and 5‐HT‐induced concentration‐dependent contractions were not statistically different between SS and SR groups (Figure 3b,c). Furthermore, pretreatment of MRAs with L‐NAME did not alter the maximum response to 5‐HT in either SS or SR groups (Figure 3c,d).

In endothelial‐intact MRA rings precontracted with PE, Ach induced a concentration‐dependent relaxation in both SS and SR groups (Figure 3e). However, the effects were significantly decreased in the SS group (*E*_max_: −71 ± 16%, −96 ± 5%, for SS and SR groups, respectively). Notably, pretreatment of aortic rings with L‐NAME almost completely abolished Ach‐induced relaxation in both SS and SR groups (Figure 3f).

As shown in Figure 3g, in endothelial‐intact MRA rings precontracted with PE, SNP induced a concentration‐dependent relaxation in both SS and SR groups. While there was no difference in the maximum response between both groups, the concentration‐response curve was moderately shifted to the left in the SS group, suggesting increased sensitivity (EC_50_: −8.15 ± 0.15, −7.7 ± 0.12; for SS and SR groups, respectively).

### 3.4 Vascular reactivity in MRA of SHR versus WKY male rats

**FIGURE 4:**
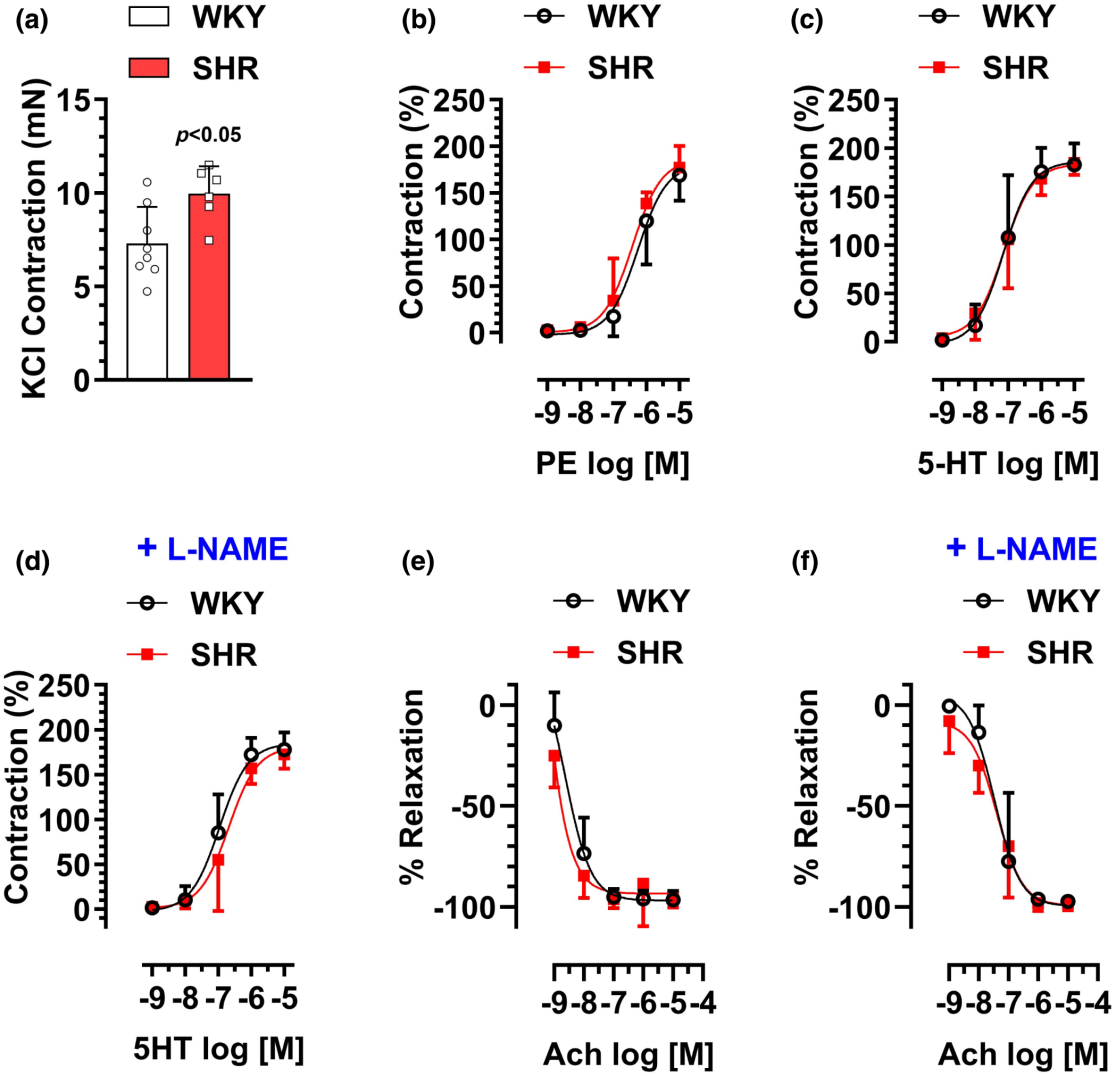
16‐ to 20‐week‐old male SHR or WKY rats were sacrificed for vascular reactivity analysis in mesenteric arterial rings. (a) Contraction induced by KCl (120 mM). (b) Contractions induced by PE (10^−9^–10^−5^ M). (c) Contractions induced by 5‐HT (10^9^–10^−5^ M). (d) Effects of pretreatment with L‐NAME (100 μM, 30 min) on 5‐HT‐induced contractions. (e) Relaxations induced by Ach (10^−9^–10^−5^ M) after precontraction with PE (10^−6^ M). (f) Effects of pretreatment with L‐NAME (100 μM, 30 min) on Ach‐induced relaxation. *N* = 6–8.

Next, we tested whether SHR rats exhibit features of vascular dysfunction in MRA segments similar to those observed in the SS rats. Similar to the SS rats, high‐KCl solution‐evoked contraction was significantly increased in the SHR group (10 ± 1.5 mN, 7.3 ± 1.9 mN, for SHR and WKY groups, respectively) (Figure 4a). When normalized to high‐KCl responses, both PE‐ and 5‐HT‐induced concentration‐dependent contractions were not statistically different between SHR and WKY groups (Figure 4b,c). Furthermore, pretreatment of MRAs with L‐NAME did not alter the maximum response to 5‐HT in either SHR or WKY groups (Figure 4d).

In endothelial‐intact MRA rings precontracted with PE, Ach‐induced concentration‐dependent relaxations were not statistically different between SHR and WKY groups (Figure 4e). While pretreatment of aortic rings with L‐NAME did not alter the maximum relaxation response to Ach in either SHR or WKY groups (Figure 4e,f), the concentration‐response curves were markedly shifted to the right in both groups (EC_50_: −7.4 ± 0.2, −8.8 ± 0.4; for SHR group, in the presence or absence of L‐NAME, respectively, respectively; EC_50_: −7.5 ± 0.2, −8.6 ± 0.2; for WKY group, in the presence or absence of L‐NAME, respectively).

### 3.5 Histological analysis of MRA

**FIGURE 5:**
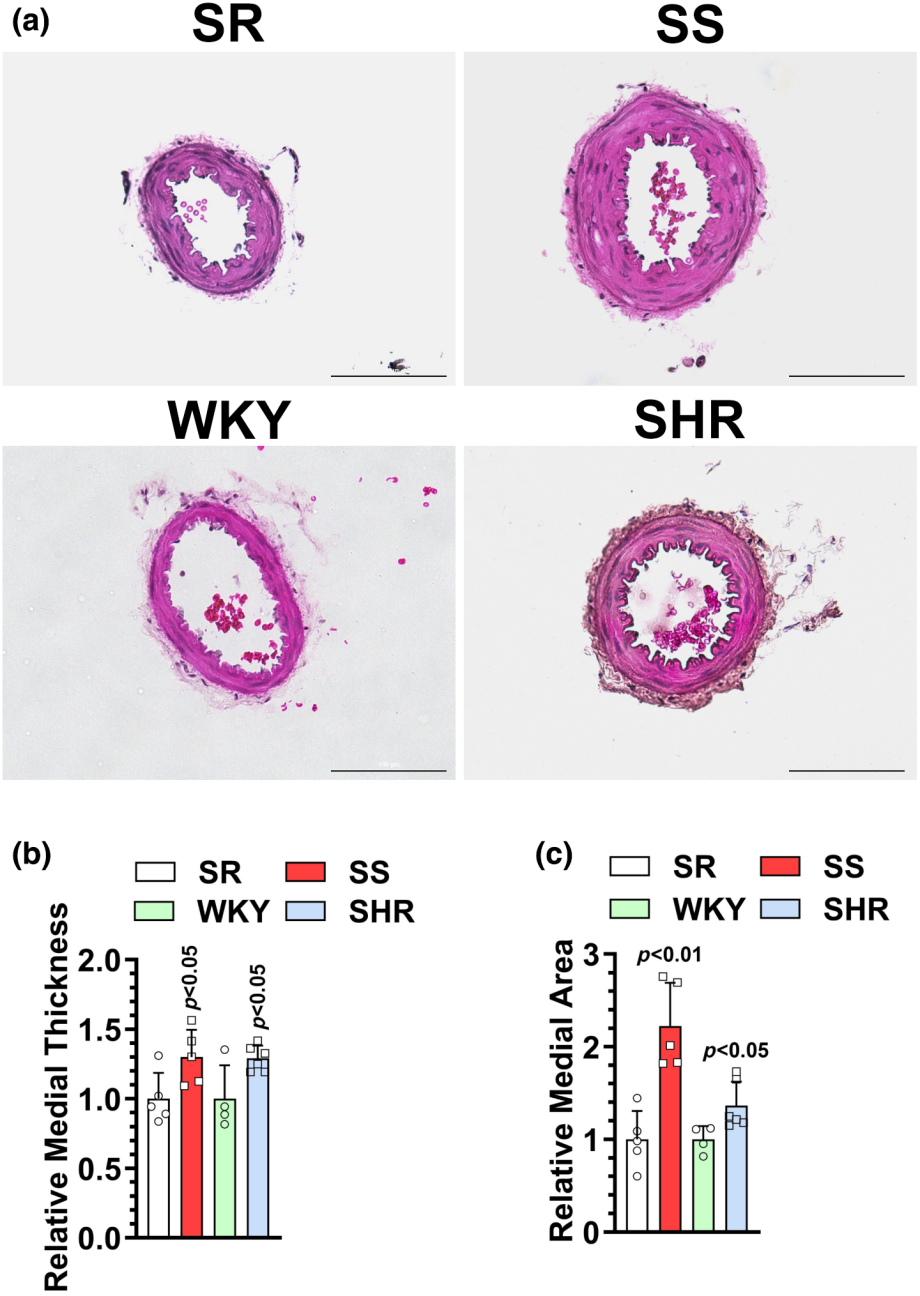
16‐ to 20‐week‐old male SS, SR, SHR, or WKY rats were sacrificed for histological analysis in mesenteric arterial rings. (a) Representative hematoxylin and eosin‐stained images (scale bar = 100 μm). (b) Relative medial thickness. (c) Relative medial cross‐sectional area. *N* = 5.

Next, we evaluated whether the enhanced responsiveness to different vasoconstrictors observed in MRA of the S rat (Figure 3) and the SHR (Figure 4) is associated with vascular remodeling. As shown in Figure 5, both S and SHR MRAs exhibited significant increases in medial wall thickness and cross‐sectional area consistent with vascular remodeling.

## 4 DISCUSSION

Conduit and resistance vessels exhibit many striking differences including varied responses to vasoactive substances under different physiological and pathophysiological states. Furthermore, they have different roles in the etiology of hypertension. Accordingly, in this study we characterized the vascular reactivity in adult male SS/Jr versus SR/Jr, maintained on a low‐salt diet (0.3%), utilizing both a conduit vessel (dorsal aorta) and a resistance artery (mesenteric resistance artery). We also performed similar experiments in age‐ and sex‐matched SHR versus WKY groups. Below, we discuss the observed similarities and differences in vascular responses between these two experimental models of hypertension, then we describe how our findings compare with previously published material.

In the dorsal aorta, we observed several similarities between the SS and SHR rats when compared to their respective controls. First, while high‐KCl solution evoked responses in both SS and SHRs that were not different from their respective controls, both animal models exhibited enhanced vasocontractile responses to 5‐HT, consistent with hyperresponsiveness of blood vessels to 5‐HT in hypertensive animal models (Banes & Watts, 2002; Huzoor et al., 1989; McGregor & Smirk, 1970). Second, the normotensive controls, SR and WKY rats, exhibited an expected increase in 5‐HT vasocontractile responses in the presence of an eNOS inhibitor, L‐NAME, demonstrating a preserved basal NO release/function. Conversely, in both SS and SHRs, 5‐HT responses were insensitive to L‐NAME pretreatment, which suggests attenuated basal release and/or bioavailability of NO in the aortic vascular wall in both these animal models, a common feature of endothelial dysfunction (Vanhoutte et al., 2017). Third, in both SS and SHR animal models, higher concentrations of Ach led to vasoconstriction, which in the case of SS rats, was moderate and was only evident in the presence of an eNOS inhibitor. Notably, Ach‐mediated vasoconstriction in both animal models was blocked by either Indomethacin (a nonselective COX inhibitor) or Terutroban (a thromboxane/prostaglandin endoperoxide receptor antagonist), suggesting it is mediated by the release of a COX‐dependent vasoconstrictive factor(s), as previously reported in high‐salt‐fed SS rats (Zhou et al., 1999, 2001) and SHRs (Konishi & Su, 1983; Lüscher & Vanhoutte, 1986; Sawada et al., 1994).

In mesenteric arteries, we also observed several similarities between SS and SHRs when compared to their respective controls. First, both animal models exhibited increased responses to multiple vasoconstrictors including the depolarizing high‐KCl solution, PE, and 5‐HT. In both animal models, when PE and 5‐HT concentration‐response curves were normalized to high‐KCl solution responses, the enhanced contractile responses in the SS and SHRs were completely abolished. This suggests a state of hypercontractility that is not specific to a particular agonist and is likely a consequence of vascular remodeling (Figure 5) due to either hypertrophy or hyperplasia (d'Uscio et al., 1997; Heagerty et al., 1993; Lee et al., 1983; Warshaw et al., 1979). Second, pretreatment of mesenteric arteries with L‐NAME did not enhance the responses to 5‐HT in SS, SHR, or even their respective controls. This suggests that unlike in the dorsal aorta, where the basal release of NO plays a key role in the regulation of vascular tone, the basal release of NO seems to play an insignificant role in the regulation of vascular tone in mesenteric arteries of SS, SR, SHR, or WKY rats. Another possible explanation for these unexpected observations is that we compared 5‐HT concentration‐response curves using the same vascular segments in the absence and then the presence of L‐NAME. This “sequential experimental design” is expected to cause some decrease in response in the latter dose—response curve due to receptor desensitization upon repeated exposure to high concentrations of 5‐HT, which may have diminished 5‐HT responses in the presence of L‐NAME (Schubert et al., 2023). Nonetheless, since we used the same experimental approach in the dorsal aorta, where we observed a marked increase in 5‐HT response in the normotensive controls, in the presence versus the absence of L‐NAME, we conclude that the role of basal NO release in the regulation of vascular tone is more evident in the dorsal aorta versus MRA in SR and WKY rat strains. Furthermore, while Ach induced varied responses in the mesenteric arteries of SS versus SHRs (discussed below), higher Ach concentrations did not cause vasoconstriction in either model, even in the presence of L‐NAME, which is compatible with the findings that COX‐derived vasoconstrictors are formed from arachidonic acid mainly in great arteries (Félétou et al., 2011).

In contrast to the similarities between the SS and SHR described above, we observed several key differences that are discussed below. First, in the dorsal aorta of SS rats, we observed a marked decrease in phenylephrine‐mediated response in contrast to a trend for an increase in the maximum response in the SHR (*p* = 0.07). Second, while both SS and SHR rats exhibited endothelial dysfunction characterized by loss of basal NO release/bioavailability, SHR still exhibited enhanced responses to 5‐HT even when eNOS was inhibited, suggesting that both endothelial‐ and SMC‐mediated mechanisms are responsible for the enhanced sensitivity to 5‐HT in SHR aorta. These SMC‐mediated mechanisms may also explain the trend for an increase (*p* = 0.07) in the maximum phenylephrine‐mediated response observed in the SHR, but not the SS rats. Third, features of vascular dysfunction in the dorsal aorta varied between SS and SHR animal models. In SS rats (Figure 1e–h), Ach caused a major NO‐dependent relaxation at all concentrations used, which was attenuated by COX‐dependent contraction at concentrations 10^−6^ M or higher. In contrast, in SHR (Figure 2e–h), Ach caused relaxation only at 10^−8^ and 10^−7^ M concentrations, whereas 10^−6^ and 10^−5^ M concentrations caused marked COX‐dependent contraction of aortic rings. Furthermore, In SS rats, Ach‐dependent maximum relaxation was markedly lower than the respective controls, which suggests attenuated eNOS activity. On the other hand, in the SHR dorsal aorta, the maximum relaxation induced by Ach did not vary versus WKY controls. Furthermore, the endothelial‐independent NO donor, SNP, induced less relaxation in SS rats, suggesting SMC‐specific dysfunction in relaxation mechanisms. Together, these observations suggest that vascular dysfunction in the SS dorsal aorta comprises attenuated eNOS activity, enhanced production of COX‐derived vasoconstrictive factor(s), and attenuated SMC‐specific relaxation mechanisms. While endothelial dysfunction in the SHR dorsal aorta is mainly due to the enhanced production of COX‐dependent vasoconstrictive factor(s).

Several key differences were also noted in the mesenteric arteries of SS versus SHR strains. While endothelial dysfunction was evident in SS rats, supported by attenuated Ach‐dependent relaxation, surprisingly, endothelial function seemed to be preserved in the SHR. More strikingly, we observed a marked deviation between the contributary mechanisms that mediate Ach‐dependent relaxation in rat models that were initially derived from outbred Sprague–Dawley (i.e., SR and SS) versus Wistar (i.e., WKY and SHR) rat strains. In Sprague–Dawley‐derived rat strains, L‐NAME almost completely abolished Ach‐dependent relaxation, suggesting predominantly NO‐dependent mechanisms. Conversely, in Wistar‐derived rat strains, L‐NAME caused a right shift in Ach concentration‐response curves without altering the maximum response, suggesting that in addition to NO, NO‐independent mechanisms, likely mediated by endothelium‐derived hyperpolarizing factor(s) and/or prostacyclin, play a key role. Notably, our observations in WKY and SHRs are consistent with previous studies demonstrating the contribution of all three vasodilator systems (NO, prostacyclin, and endothelium‐derived hyperpolarization) in Ach‐mediated relaxation in isolated mesenteric arteries of WKY and SHRs (Jiang et al., 2016) and in vivo (Behuliak et al., 2011). However, in contrast to our observations in SR and SS rats demonstrating a predominant role for NO‐dependent mechanisms in mediating Ach‐mediated relaxation in mesenteric arteries, previous studies utilizing conscious rats in vivo demonstrated that NO deficiency in SS/Jr rats is accompanied by a compensatory increase in NO‐independent systems that contributed to BP regulation (Behuliak et al., 2011). Together, these observations suggest that this reported compensatory increase in NO‐independent systems may therefore be more relevant for vascular beds other than mesenteric arteries in vivo.

Under physiological conditions, the vascular endothelium secretes several relaxing and contracting factors that tightly regulate vascular tone. However, in many cardiovascular diseases, such as HTN and atherosclerosis, the endothelium becomes dysfunctional, which is characterized by decreased release/activity of endothelium‐derived relaxing factors (EDRF) and simultaneous augmented release/activity of endothelium‐derived contracting factors (EDCF). In conduit vessels of the SHR, endothelial dysfunction has been attributed mainly to the enhanced release of COX‐dependent EDCF(s) rather than a decreased release of NO (Boulanger, 1999; Konishi & Su, 1983; Lüscher & Vanhoutte, 1986; Sawada et al., 1994). Furthermore, mesenteric resistance vessels from SHRs exhibit vascular remodeling that is expected to cause hyperresponsiveness to vasocontractile agonists (Heagerty et al., 1993; Lee et al., 1983; Warshaw et al., 1979). As discussed above, our results in the SHR model are, in general, consistent with these prior observations. However, our study failed to demonstrate any evidence of endothelial dysfunction in SHR mesenteric arteries, which could be related to age, source, substrain, or other methodical differences between experimental procedures. Consistent with this notion, previous studies demonstrate that Ach‐mediated relaxation was maintained in mesenteric arteries of 12‐week‐old SHR versus WKY rats, but progressively declined with aging (Kong et al., 2015).

In SS rats fed a high‐salt diet, previous studies demonstrate that they exhibit endothelial dysfunction that is attributed to both impaired activity of eNOS (Boulanger, 1999; Hayakawa & Raij, 1997; Lüscher et al., 1987) and enhanced release of COX‐dependent EDCF(s) (Zhou et al., 1999, 2001). In addition, SS rats fed a high‐salt diet exhibit impaired SMC‐dependent relaxation mechanisms (Zhou et al., 2000) and microvascular remodeling (d'Uscio et al., 1997). Notably, the SS rat substrains used in these previous studies typically required high‐salt feeding to elicit HTN, and, commonly, vascular dysfunction was not observed in SS rats on a low‐salt diet (Hayakawa et al., 1997; Kong et al., 1991; Lüscher et al., 1987; Raij et al., 1988; Zhou et al., 1999, 2001). In contrast, in this study, we observed several key features of vascular dysfunction in SS rats maintained on a low‐salt diet including vascular endothelial dysfunction and hypercontractility, which are reminiscent of vascular changes observed in SS rats with high‐salt feeding. The apparent disagreement between our observations and prior studies that reported lack of vascular dysfunction in SS rats on a low‐salt diet is likely attributed to the different substrains of SS/Jr rats used across these studies, which have been shown to exhibit genetic (Padmanabhan & Joe, 2017; Pai et al., 2018) and phenotypic variability (Rapp & Garrett, 2019).

In conclusion, this study provides evidence to support that the male SS/Jr rat strain maintained on a low‐salt diet could be utilized as a valid animal model for recapitulating vascular dysfunction and that while high‐salt feeding may exacerbate vascular dysfunction in the SS/Jr rat, is not required to induce it. Our study also suggests that during the early development of the SS/Jr rat, susceptibility alleles for vascular dysfunction were selected for along with those responsible for hypertension and salt sensitivity, and thus this model may be useful in the identification of novel genes and pathways that cause vascular dysfunction.

## AUTHOR CONTRIBUTIONS

I.O. conceived and designed research; A.G., S.K., and B.M. performed experiments; I.O., A.G., and S.K. analyzed data; I.O. interpreted results of experiments, prepared figures, and drafted the manuscript; B.J. provided the SS/Jr model from the colony maintained at our university. A.G., S.K., B.M., J.Z., B.J., and I.O. edited and revised the manuscript, and approved the final version of the manuscript.

## FUNDING INFORMATION

This work was supported by NHLBI awards (R00 HL153896 to I.O. and R01 HL152162 to J.Z.).

## CONFLICT OF INTEREST STATEMENT

No conflicts of interest, financial or otherwise, are declared by the authors.

## ETHICS STATEMENT

The study was performed per the University of Toledo Health Science Campus Institutional Animal Care and Use guidelines.

## Data Availability

Data will be made available as stipulated in the sharing of data section of the NIH grant supporting this work.
